# Avoiding drug resistance through extended drug target interfaces: a case for stapled peptides

**DOI:** 10.18632/oncotarget.8572

**Published:** 2016-04-04

**Authors:** Siau Jia Wei, Sharon Chee, Larisa Yurlova, David Lane, Chandra Verma, Christopher Brown, Farid Ghadessy

**Affiliations:** ^1^ P53 Laboratory (A*STAR), #06-04/05 Neuros, 138648, Singapore; ^2^ Bioinformatics Institute (A*STAR), 07-01 Matrix, 138671, Singapore; ^3^ ChromoTek GmbH, 82152 Planegg-Martinsried, Germany; ^4^ School of Biological Sciences, Nanyang Technological University, 637551, Singapore; ^5^ Department of Biological Sciences, National University of Singapore, 117543, Singapore

**Keywords:** p53, HDM2, MDM2, stapled peptide, cancer resistance

## Abstract

Cancer drugs often fail due to the emergence of clinical resistance. This can manifest through mutations in target proteins that selectively exclude drug binding whilst retaining aberrant function. *A priori* knowledge of resistance-inducing mutations is therefore important for both drug design and clinical surveillance. Stapled peptides represent a novel class of antagonists capable of inhibiting therapeutically relevant protein-protein interactions. Here, we address the important question of potential resistance to stapled peptide inhibitors. HDM2 is the critical negative regulator of p53, and is often overexpressed in cancers that retain wild-type p53 function. Interrogation of a large collection of randomly mutated HDM2 proteins failed to identify point mutations that could selectively abrogate binding by a stapled peptide inhibitor (PM2). In contrast, the same interrogation methodology has previously uncovered point mutations that selectively inhibit binding by Nutlin, the prototypical small molecule inhibitor of HDM2. Our results demonstrate both the high level of structural p53 mimicry employed by PM2 to engage HDM2, and the potential resilience of stapled peptide antagonists to mutations in target proteins. This inherent feature could reduce clinical resistance should this class of drugs enter the clinic.

## INTRODUCTION

Stapled peptides are emerging as a robust class of synthetic biologics capable of selectively perturbing protein-protein interactions (PPIs) [1, 2]. The judicious introduction of a chemical tether (the “staple”) linking two amino acid side chains of a peptide can result in pre-stabilization of the alpha helical conformation favoured in complex formation with a target protein. Importantly, stapling can also impart the desirable drug-like properties of cell penetration, protease resistance and intracellular target engagement on otherwise biologically inert peptides [3, 4]. Stapled peptides targeting numerous intracellular targets have been described, with many employing an all-hydrocarbon olefin stapling moiety [5]. Alternative stapling chemistries have also been reported [6, 7].

The stapled peptide PM2/sMTide-02 binds to the highly evolutionary conserved E3 ubiquitin ligase HDM2 [3, 8, 9]. PM2 inhibits HDM2 from targeting p53 for proteosomal degradation via ubiquitination [10–13]. Cell fate in response to a plethora of stress signals is directed by p53 [14, 15]. The pro-apoptotic activity of p53 is compromised in 50% of all cancers via mutation, highlighting its importance [16, 17]. In certain cancers where p53 is not mutated, its function is often mitigated through overexpression of HDM2 [18, 19]. Inhibition of HDM2 by a wide range of antagonists has been shown to lead to increased cellular levels of wild type p53 and cell death [20–23]. In normal cells, HDM2 inhibition can cause reversible arrest, highlighting a possible target for cyclotherapy regimens [24–26]. The prototypical HDM2 antagonist Nutlin and its numerous derivatives comprise a signature chemotope that recapitulates the interaction of 3 key residues in the N-terminal region of p53 (F19, W23, L26) with a hydrophobic pocket in the N-terminal domain of HDM2 [20, 27–29]. These 3 residues are absolutely required in effective peptidic antagonists including PM2 [30–32].

*In vitro* selection has identified point mutations in HDM2 that selectively abrogate Nutlin binding, with no loss in interaction with p53 [33]. As small molecule HDM2 inhibitors have only recently entered clinical trials [34–40], it remains to be seen whether this mechanism of drug resistance will be realized in patients with cancers that retain wild-type p53. *Ex vivo* studies have indicated inactivating p53 mutations and endoreduplication as principal modes of resistance to Nutlin efficacy [38, 41–43]. However, a recent *in vivo* study using xenograft tumours in mice showed development of resistance to the Nutlin analogue SAR405838 was associated with a point-mutated p53 that still retained activity [23, 44]. Notably, PM2 and several derivatives are able to bind and antagonize Nutlin-resistant HDM2 [45]. This is attributed to the broad, diffuse network of contacts they form with HDM2, which contrasts with the intrinsically limited number of “anchor” points employed by the comparatively small molecule Nutlin [20, 46, 47].

The engagement mode of peptidic antagonists suggests that resistance through point mutation in target proteins is less likely compared to small molecule binders. However, this has yet to be experimentally verified. Here, using the PM2-HDM2 interaction as a model system, we carried out *in vitro* selections to identify point mutations in the N-terminal domain of HDM2 that would selectively preclude binding of PM2 but not p53. The results show that a significant phenotype is only commensurate with co-loss of p53 binding, and therefore unlikely to occur in cancers that retain p53 function. Peptidic drugs may therefore prove robust antagonists in oncology applications, where clinical resistance is of fundamental importance to the treatment outcome [48, 49].

## RESULTS

### HDM2 variants resistant to PM2 inhibition show reduced p53 binding

**Figure 1:**
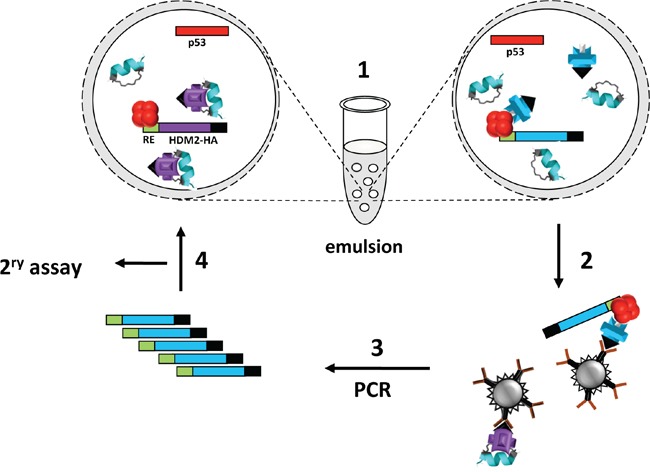
Selection of PM2-resistant HDM2 by *in vitro* compartmentalisation **1.** HDM2 expression constructs (blue and purple bars) appended with 2CONA p53 response element (“RE”, green) and HA-tag coding sequence (black) and p53 expression construct (red bar) are segregated into aqueous emulsion compartments along with PM2 (cyan helix). Protein expression occurs within compartments. PM2 inhibition of HDM2 results in no HDM2-p53-DNA complex formation (left bubble), whereas resistant HDM2 can form the complex (right bubble). **2–3**. The emulsion is broken and complexes captured with anti-HA antibody. DNA encoding resistant HDM2 variants is amplified by PCR. **4.** Selectants further evaluated by secondary pull-down assay or subjected to further rounds of selection.

**Figure 2:**
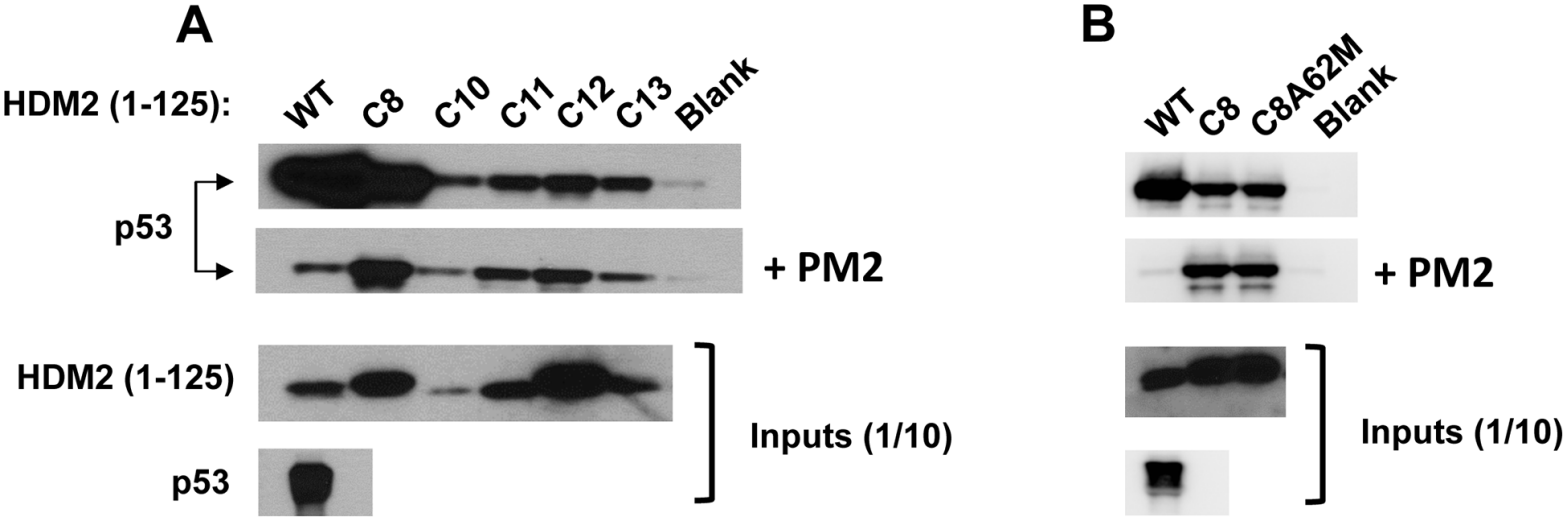
Selected HDM2 variants display *in vitro* PM2-resistance phenotype **A.** *In vitro* pull-down assay showing reduced inhibition by PM2 (10 μM) to binding of p53 for indicated parental HDM2 variants and WT HDM2 (residues 1-125). Note: exposure time for HDM2 inputs is 8 hours and 1 second for all other panels. **B.** *in vitro* pull-down assay showing little impact upon reversion of the M62A mutation to PM2 binding in HDM2-C8 (residues1-125). Blank indicates background p53 binding in absence of HDM2. Note: exposure time for HDM2 inputs (developed using film) is 8 hours and 10 second for all other panels (digitally acquired).

**Figure 3:**
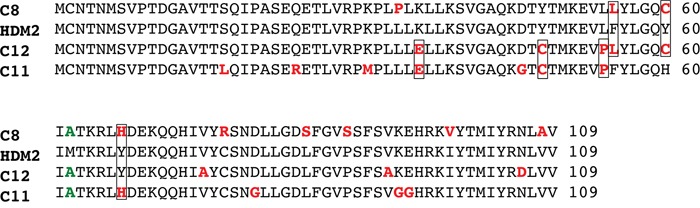
Sequence alignment of selectant HDM2 clones showing PM2 resistance Mutated residues are highlighted in red, with those present in more than one selectant boxed. The M62A mutation (green) was incorporated into the selection library.

**Figure 4:**
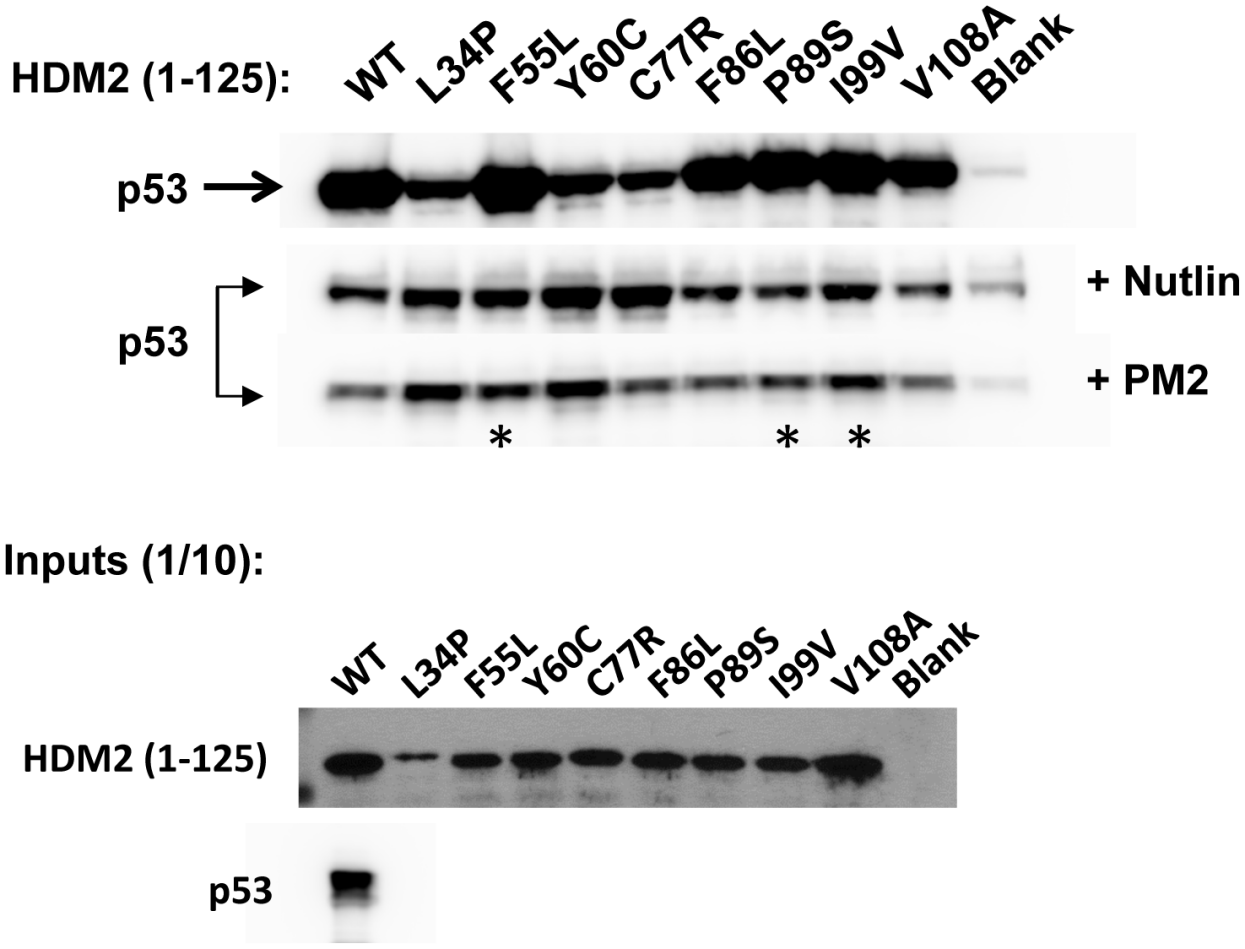
PM2 resistance comes at cost of reduced interaction with p53 *In vitro* pull-down assay showing reduced inhibition by PM2 (10 μM) to indicated point mutants (asterisk) derived from HDM2-C8 (residues 1-125). The point mutants L34P, F55L, Y60C and C77R also show reduced inhibition by Nutlin (10μM). Blank indicates background p53 binding in absence of HDM2. Note: exposure time for HDM2 inputs is 8 hours (developed using film) and 3 minutes for all other panels (digitally acquired).

**Figure 5:**
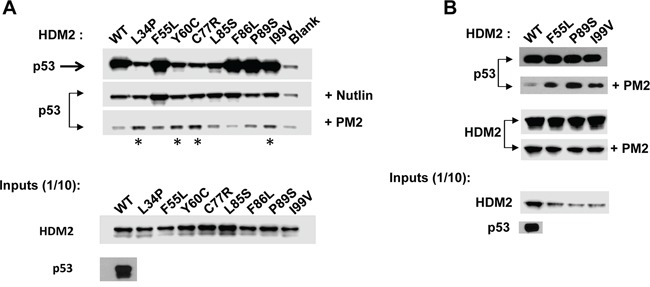
PM2 resistance is also seen when point mutants are introduced into full-length HDM2 **A.** *In vitro* pull-down assay showing reduced inhibition by PM2 (10 μM) to indicated C8-derived point mutants (asterisk) present in full-length HDM2 The point mutant F55L also shows reduced inhibition by Nutlin (10μM). Blank indicates background p53 binding in absence of HDM2. **B.** As in A, additionally showing levels of wild type and indicated HDM2 variants co-eluted off beads after pull-down following mock (panel 3) and PM2 treatment (panel 4). Note exposure time for p53 pull-down in absence of treatment (panel 1) is 5s and 10 minutes for pull-down after PM2 treatment (panel 2, developed using film). Exposure time for HDM2 input and HDM2 (+ indicated variants) eluted off beads after pull-down is 30 seconds (digitally acquired).

To evolve PM2-resistant HDM2 we used a previously described method that enabled selection of Nutlin-resistant HDM2 variants (Figure 1) [33, 50, 51]. A library of randomly mutated genes expressing the HDM2 N-terminal domain (with a C-terminal HA tag) and containing a p53 response element (RE) was clonally segregated into the aqueous compartments of a water in oil emulsion along with the p53-expressing gene cassette and PM2. Within each compartment, protein expression occurs, and in the absence of inhibitor, a complex forms between p53, variant HDM2 and the gene encoding the variant HDM2. In the presence of PM2, this complex does not form unless the HDM2 is mutated to exclude PM2, but not p53 binding. Upon disruption of the emulsion, persisting complexes are enriched by immunoprecipitation using magnetic beads coated with anti-HA antibody, and the genes encoding resistant HDM2 variants amplified by PCR for further rounds of selection and/or secondary assays. After 4 rounds of selection, 3 HDM2 variants (C8, C11 and C12) were identified that showed PM2 resistance as judged by pull-down assay using *in vitro* expressed proteins (Figure 2A). Whilst these appeared significantly resistant to PM2, with little or no reduction in their interaction with p53 in the presence of PM2 (top and second panel), this came at the cost of reduced p53 binding compared to wild type N-terminal domain, particularly for C11 and C12. All selectants showed a high mutational burden, with 9-12 mutations present in each (Figure 3). Six specific mutations were present in more than one selectant (boxed), highly indicative of positive selection. The initial library was made to include the M62A mutation shown to abrogate Nutlin binding. Whilst this mutation in isolation does not affect PM2 binding, it was introduced to bias selections as it removes a sizeable packing interface between PM2 and HDM2 [47]. However, reversion of this mutation in the C8 selectant did not alter the phenotype (Figure 2B), indicating the importance of the other mutations. The C8 selectant showed the strongest resistance phenotype, and therefore all 9 constituent mutations were next analysed as N-terminal single point mutants to assay their relative contributions (Figure 4 and Figure S1A). The mutations generally fell into two groups: a subset that was clearly resistant to PM2 binding albeit at the cost of reduced p53 binding (L34P, Y60C) and a group that retained p53 binding and showed weak resistance to PM2 (F55L, P89S, I99V). Interestingly, with the exception of I99V, all of these mutants displayed Nutlin resistance (Figure 4, panel 2). The Y67H, C77R, and V108A point mutants showed no PM2-resistance phenotype. The reduced binding of the L34P mutant may arise from the significantly reduced expression levels in the assay compared to WT N-terminal domain (Figure 4). However, reduced p53 interaction was also observed in subsequent experiments using the full-length HDM2 point mutant whose expression was comparable to WT, both *in vitro* and *ex vivo* (Figure 5, 6B). The mutations displaying the PM2-resistant phenotype behaved similarly when introduced as point mutants into full-length HDM2 and assayed for p53 interaction (Figure 5A, 5B). In the context of full-length protein, only the F55L mutant showed notable Nutlin resistance (Figure 5A). The difference in behavior towards Nutlin binding/inhibition between the N-terminal and full-length proteins possibly results from the secondary p53 interaction site in the acidic domain of HDM2 [52, 53].

We additionally analysed the K36E, Y48C and L54P point mutants derived from the C11 and C12 selectants as these mutations were shared exclusively between them. Only the L54P mutation showed an exceptionally weak resistance phenotype, and this came at the cost of reduced interaction with p53 (Figure S1B).

### HDM2 variants show weak resistance phenotype in DKO cells

**Figure 6:**
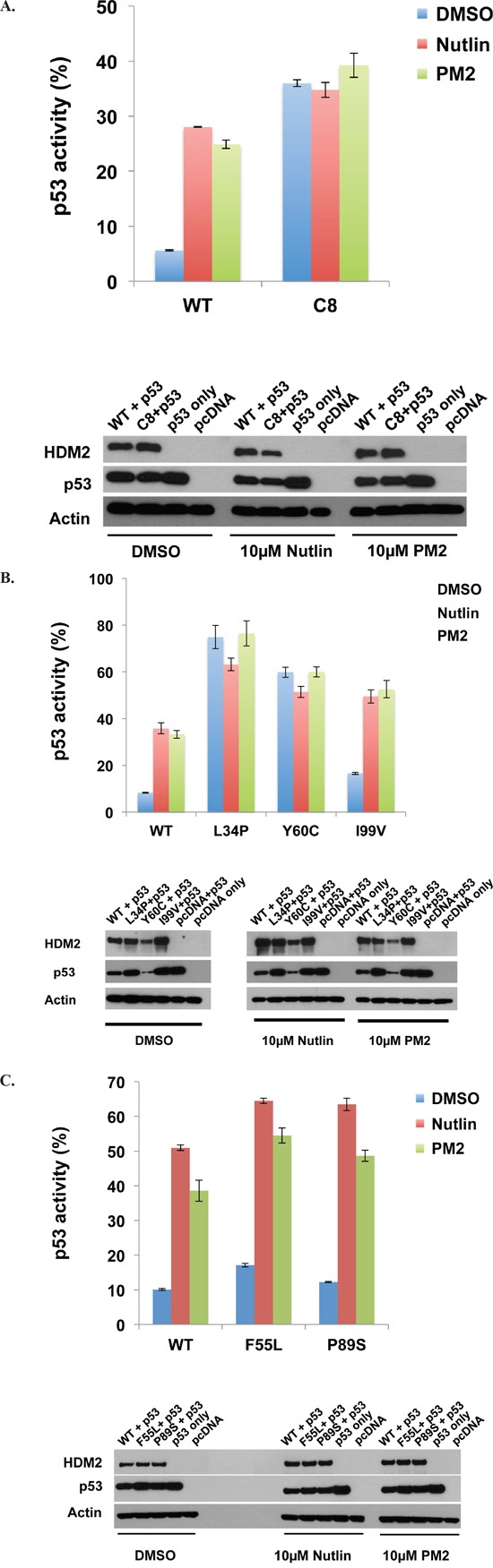
PM2 shows reduced inhibition of selected HDM2 variants in p53/MDM2-null DKO cells **A.** Wild-type and HDM2-C8 (full-length) were co-transfected with p53 and p53-reporter gene, and reporter gene activity measured in the presence of PM2 (20 μM) or Nutlin (10 μM). p53 activity is denoted as percentage of that observed when p53-alone co-transfected with reporter gene. Shown below are Western blots indicating expression levels of HDM2 variants and p53 cotransfected into DKO cells. **B** and **C.** As in ‘A’, with wild-type HDM2 and indicated HDM2-C8 derived point mutants (full length) co-transfected into DKO cells. p53 activity is denoted as percentage of that observed when p53-alone co-transfected with reporter gene. Shown below are Western blots indicating expression levels of HDM2 variants and p53 cotransfected into DKO cells.

We next investigated the behavior of the C8 HDM2 variant *ex vivo*, using p53/MDM2-null DKO cells. Plasmids encoding p53, HDM2 and a p53-reporter gene were transfected into cells, and transactivation by p53 measured. HDM2 ablated p53 activity to ~ 6% of that observed in the absence of HDM2 co-transfection (Figure 6A). Addition of Nutlin or PM2 inhibited HDM2, with p53 activity rising to ~26% for both. In the case of HDM2-C8, initial knockdown of p53 activity was ~ 6-fold reduced, most likely a result of the reduced interaction capability of C8 with p53 (Figure 2). Importantly, addition of PM2 did not result in any significant increase in p53 activity, indicating a resistance phenotype. Similarly, Nutlin had little effect in abrogating the function of C8. Analysis of C8-derived point mutants showed that the L34P and Y60C mutations in isolation could partially recapitulate the parental phenotype (Figure 6B). Both mutants showed significantly decreased knockdown of p53 activity (~7 fold compared to WT HDM2), again most likely due the reduced interaction observed *in vitro* (Figures 4, 5), with no rescue observed after addition of either PM2 or Nutlin. The F55L and I99V point mutants displayed a weak resistance phenotype in this assay. Both reduced knockdown of p53 activity (~1.5-fold compared to WT HDM2) and whilst addition of PM2 rescued activity, the magnitude of this was consistently less for both mutants compared to WT HDM2 (~3.2 versus ~ 4-fold)(Figures 6B, 6C). The P89S mutant essentially behaved like wild-type HDM2.

### The HDM2-F55L variant shows reduced relative binding affinity to PM2

We next used the fluorescence polarisation (FP) assay to measure relative binding affinity of PM2 to recombinantly expressed HDM2 (1-125) and relevant mutants. Only the F55L mutant could be stably expressed in *E. coli* and purified, and this showed a slightly reduced relative binding affinity to PM2 compared to WT HDM2 (117 ± 30 nM and 53 ± 9 nM respectively), consistent with the *in vitro* pull-down assays and cell-based reporter assay. Using this assay, binding to the p53 peptide (amino acids 16 to 29) was also slightly comprised for F55L compared to WT HDM2 ( 7 ± 1.2 μM and 3 ± 0.7 μM respectively), in this case consistent with the slightly reduced activity of HDM2 F55L on p53 function compared to WT (Figure 6C).

### No significant resistance observed in BHK cells using the fluorescent two-hybrid assay

Interaction of HDM2-C8 and the L34P, F55L, Y60C point mutants with p53 was further studied using the fluorescent two-hybrid assay (F2H) [54]. This assay facilitates real-time detection of p53-HDM2 interaction in living cells and perturbation by small molecule/peptidic antagonists. It requires expression of the p53 N-terminal domain (residues 1-81) as a fusion with GFP and HDM2 (residues 7-134) as a fusion with RFP in transgenic F2H-BHK mammalian cells. As observed for recombinant expression in *E.Coli*, only the HDM2-F55L mutant could be stably expressed in this system, and the results indicated no significant difference compared to WT for inhibition by both PM2 and Nutlin (Figure S2). The weak difference observed in pull-down, reporter and FP assays is therefore likely to be below the detection threshold of the F2H assay or to be less profound in an intracellular environment. Collectively, these results confirm what is essentially a weak phenotype for the F55L mutation in mammalian cells.

## DISCUSSION

The comparatively larger interaction footprint of a stapled peptide antagonist should impart broad resistance to point mutations. To test this hypothesis, we carried out a high-throughput selection for mutations in the N-terminal domain of HDM2 that inhibit stapled peptide, but not p53 binding. The results indicate that whilst PM2-binding can be abrogated by mutation in HDM2, this generally comes at the cost of significantly reduced p53 binding, and hence would be unlikely to occur in cancers where p53 is not frequently inactivated. Note that the residual p53 binding of these mutants is sufficient to withstand the selection conditions employed. This binding may partially result from a secondary interaction site in the C-terminal domain of p53 that interacts with the HMD2 N-terminal domain [55].

**Figure 7:**
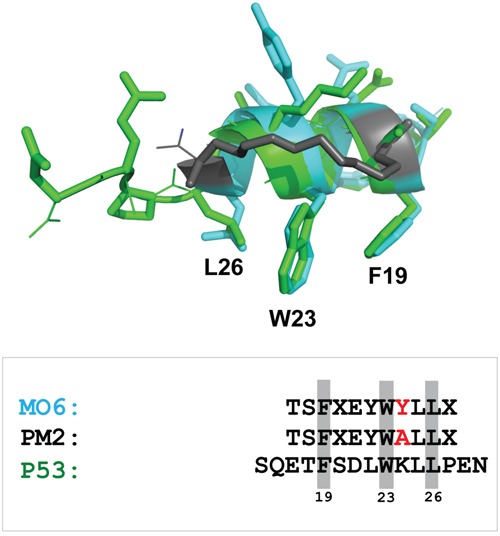
Stapled peptide recapitulates key p53 signature residues that interact with HDM2 N-terminal domain Overlay of p53 peptide (green) and MO6 stapled peptide (cyan, with staple moiety in grey) when bound to HDM2 N-terminal domains. The relative configurations of the key F19 and W23 residues are conserved, with some deviation in the orientation of L26. Adapted from 1YCR and 4UMN. Shown below is alignment of p53 peptide, PM2 and MO6, with signature residues shaded and residue differing between PM2 and MO6 highlighted in red.

**Figure 8:**
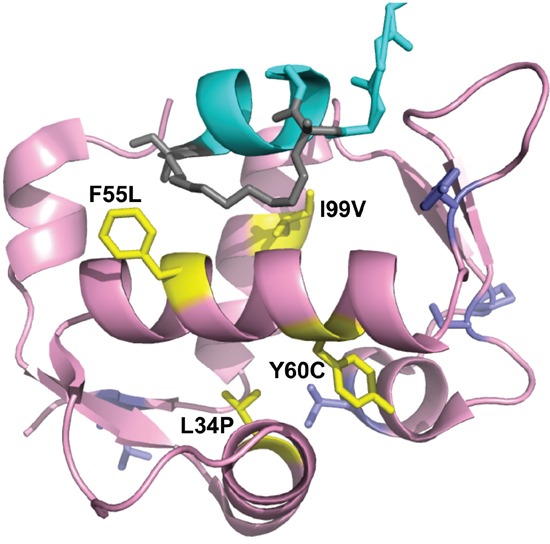
Projection of HDM2-C8 mutants onto HDM2 N-terminal domain structure Shown is structure of M06 stapled peptide (cyan, grey) bound to HDM2-M62A N-terminal domain (pink). The residues contributing to the resistance phenotype are coloured yellow, and the rest are purple. Adapted from 4UMN.

The crystal structure of the stapled peptide M06 bound to HDM2-M62A was recently described [47]. As MO6 differs from PM2 by a single amino acid (Figure 7), we have used this structure to further understand the mutations present in HDM2-C8. The mutations L34P and Y60C result in significantly decreased interaction with p53 and resistance to PM2 inhibition (Figures 4, 5, 6B). The side chains of L34 and Y60 pack against numerous hydrophobic residues contributing to the p53-binding hydrophobic cleft (Figure 8). Mutations to less bulky residues (P and C respectively) likely result in altered structural dynamics/conformation of the cleft, causing gross destabilization that inhibits both p53 and PM2 binding. Poor expression yields of proteins containing these mutations (both *in vitro* and *ex vivo*) support this notion. A very similar phenotype was observed for the L82P mutation in HDM2 that makes it resistant to Nutlin binding at the cost of reduced p53 binding [33].

**Figure 9:**
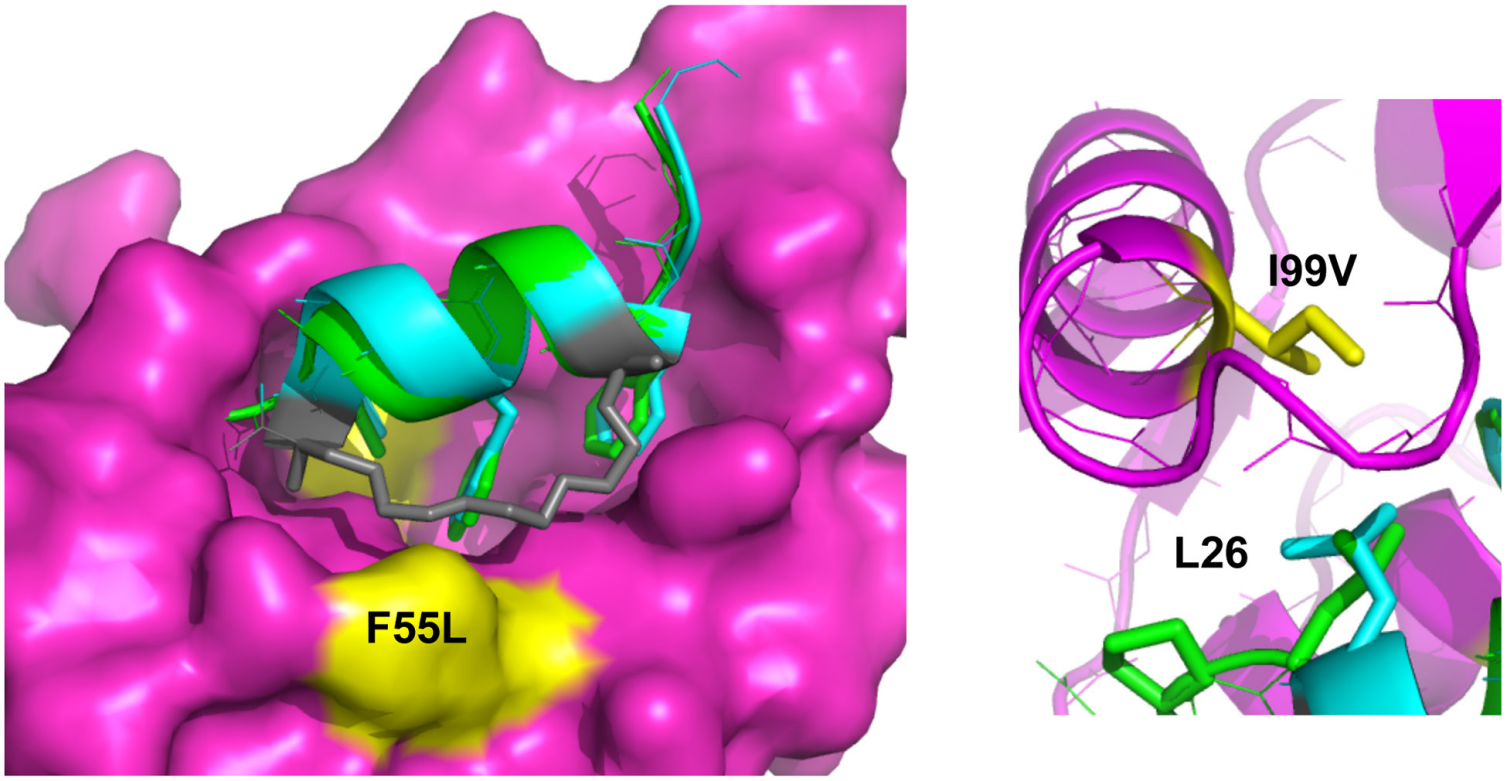
The staple moiety makes favourable contacts with F55 in the N-terminal domain of HDM2 Left: Overlay of p53 peptide (green) and MO6 stapled peptide (cyan, staple in gray) bound to HDM2 N-terminal domain (magenta, surface representation). The positions of the F55 and I99 residues are indicated in yellow. Right: Same as left, highlighting the relative orientation of the p53 peptide and MO6 stapled peptide L26 side chains in respect to I99 in HDM2.

The mutations F55L and I99V resulted in a weak PM2 resistance phenotype with p53 binding only slightly impaired (Figures 4-6). F55 resides in the α2 helix (residues 50-65) and its sidechain projects into solution. When bound to HDM2, the hydrophobic staple moiety of MO6 packs against F55 (Figure 9, left panel). The same interaction is observed in the structure of the SAH-8 stapled peptide bound to HDM2 [56]. As the hydrocarbon staple is the major differentiating factor between p53 and stapled peptide, mutations would be expected to arise that discriminate against it. Mutation to leucine could therefore result in less optimal packing of the staple against the α2 helix. To further test this, we mutated F55 to the less bulky/hydrophobic alanine and carried out *in vitro* pull down assays. Surprisingly, the results showed this mutation to make little difference towards interaction with p53 or PM2 (Figure S3). In the structure of M06 bound to HDM2-M62A the staple re-orientates itself to overcome loss of a favourable packing interaction with M62. In light of this remarkable plasticity, it is plausible that it can re-orientate to overcome loss of the favourable F55 interaction when mutated to alanine. Mutation to the slightly less bulky, but still hydrophobic leucine may not warrant conformation changes in the staple, instead resulting in slightly reduced packing interactions and the weak resistance phenotype observed.

Interaction of p53 with the HDM2 N-terminal domain is mediated by three signature residues in p53 (F19, F23, L26) that interact with discrete pockets in the HDM2 binding cleft [27]. I99 resides in the α2′ helix (residues 95-104) that forms one side of the pocket that accommodates L26 of p53. The side chain of I99 is in close proximity to L26 whose interaction is favoured through hydrophobic interactions. An overlay of the MO6 and the p53 peptides bound to HDM2 shows a relatively extended conformation of the alpha helix in the stapled peptide. This significantly alters the C-alpha position of L26, resulting in an altered side chain projection that is more proximal to I99 (Figure 9; right panel). Upon mutation to valine, the extended strand in p53 containing L26 can more readily adjust its packing arrangements to accommodate this mutation and maintain complementarity with the HDM2 protein surface. The stapled peptide is however more rigid, and has less conformational space open to it. If it were to re-orientate its binding pose to form better contacts with the valine it would likely disrupt favourable contacts elsewhere. Notably, mutation to the smaller alanine residue (I99A) did not lead to any further discrimination, resulting in drastic loss of p53 binding (Figure S4), highlighting the inability of p53 (and most likely PM2) to adjust binding pose to compensate for the loss of a critical hydrophobic interaction.

Mutations outside of the N-terminal domain of HDM2 have been shown to allosterically modulate its binding properties [33, 57]. In this study we focused on the N-terminal domain with the aim of exploring as much mutational diversity as possible within the technical confines of the *in vitro* selection platform employed (~ 10^10^ variants) [51]. It should be noted that practical limitations do not allow for interrogation of all possible mutational diversity, and that mutations conferring the desired phenotype may have been missed. However, a previous selection using the same methodology readily identified point mutations in the N-terminal domain that selectively abrogated binding by the small molecule Nutlin [33]. In light of both this observation and the exceptional structural mimicry of PM2, it appears highly unlikely that mutation in HDM2 can selectively disrupt PM2 binding. Further studies will confirm whether this observation can be extended to other peptide-protein interactions.

Synthetic peptidic ligands are being developed against a range of targets for therapeutic use. The results presented here suggest that this emerging class of drug will enable robust antagonism that is poorly ablated through mutation of the target protein. This is potentially of great relevance to the field of oncology, where clinical resistance poses significant barriers to treatment efficacy.

## MATERIALS AND METHODS

### Materials

Unless otherwise specified, all oligonucleotides used in this work were from Integrated DNA Technologies, restriction enzymes from NEB and chemical reagents from Sigma. Nutlin was from Calbiochem. Anti-HA and actin antibodies (mouse monoclonal) were purchased from Sigma. Anti-p53 antibody conjugated to horseradish peroxidase (DO1-HRP, mouse monoclonal) was purchased from Santa Cruz.

### Primers

Hdm2-Nter-noHA-R: 5′- TACTACCAAGT TCCTGTAGAT -3′

HA-F: 5′- TACCCATACGATGTTCCAGATT ACGCTTAA -3′

HDMM62A-1: 5′-CTTGGCCAGTATATTGCGACTAAACGATTATATG-3′

HDMM62A-2: 5′-CATATAATCGTTTAGTCGCAATATACTGGCCAAG-3′

petF2: 5′- CATCGGTGATGTCGGCGAT -3′

petR: 5′- CGGATATAGTTCCTCCTTTCAGCA -3′

Hdm2-NdeI: 5′- CACAACATATGTGCAATACCAACATGTCTGTACC -3′

HA-rev-BamHI: 5′- GCTCTGGATCCTTAAGCGTAATCTGGAACATCGTATGGGTA -3′

infus-Mdm2-F: 5′- AAGGAGATATACATATGTGCAATACCAACATG -3′

Infus-M62AC8-Nter-R: 5′- TACTGCCAAGTT CCTGTAGATCATGGT -3′

M62AC8Hdm2-Nter-F: 5′- ACCATGATCTACAGGAACTTGGCAGTAGTCAATCAGCAGGAATCATCGG -3′

petATG-R: 5′-CATATGTATATCTCCTTCTTAAAGTTAAAC-3′

M62AC8-revertQC1: 5′- CTTGGCCAGTGTATTATGACTAAACGATTACA -3′

M62AC8-revertQC2: 5′- TGTAATCGTTTAGTCATAATACACTGGCCAAG -3′

petF3: 5′- ATAGGCGCCAGCAACCGCACCTG -3′

mdm2-L34P-QC1: 5′- GGTTAGACCAAAGCCATTGCCTTTGAAGTTATTAAAGTCTGTTGGTGC -3′

mdm2-L34P-QC2: 5′- GCACCAACAGACTTTAATAACTTCAAAGGCAATGGCTTTGGTCTAACC-3′

mdm2-F55L-QC1: 5′- CCTATACTATGAAAGAGGTTCTTCTTTATCTTGGCCAGT -3′

mdm2-F55L-QC2: 5′- ACTGGCCAAGATAAAGAAGAACCTCTTTCATAGTATAGG -3′

mdm2-Y67H-QC1: 5′- ATGACTAAACGATTACATGATGAGAAGCAACAACATATTG -3′

mdm2-Y67H-QC2: 5′- CAATATGTTGTTGCTTCTCATCATGTAATCGTTTAGTCAT -3′

mdm2-C77R-QC1: 5′- CAACATATTGTATATcGTTCAAATGATCTTC -3′

mdm2-C77R-QC2: 5′- GAAGATCATTTGAACg ATATACAATATGTTG -3′

mdm2-L85S-QC1: 5′- GATCTTCTAGGAGATTcGTTTGGCGTGCCAAGC -3′

mdm2-L85S-QC2: 5′- GCTTGGCACGCCAAACgAATCTCCTAGAAGATC -3′

mdm2-P89S-QC1: 5′- GATTTGTTTGGCGTGtCAAGCTTCTCTGTGAAAGAGC -3′

mdm2-P89S-QC2: 5′- GCTCTTTCACAGAGAAGCTTGaCACGCCAAACAAATC -3′

mdm2-I99V-QC1: 5′- GCTTCTCTGTGAAAGAGCACAGGAAAgTATATACCATGATCTACAGG -3′

mdm2-I99V-QC2: 5′- CCTGTAGATCATGGTATATAcTTTCCTGTGCTCTTTCACAGAGAAGC-3′

mdm2-V108A-QC1: 5′- CCATGATCTACAGGAACTTGGcAGTATACCCATACG -3′

mdm2-V108A-QC2: 5′- CGTATGGGTATACTgCCAAGTTCCTGTAGATCATGG -3′

mdm2-I99A-QC1: 5′- GAAAGAGCACAGGAAAgcATATACCATGATCTA -3′

mdm2-I99A-QC2: 5′- TAGATCATGGTATATgcTTTCCTGTGCTCTTTC-3′

mdm2-F55A-QC1: 5′- ATGAAAGAGGTTCTTgcTTATCTTGGCCAGTA -3′

mdm2-F55A-QC2: 5′- TACTGGCCAAGATAAgcAAGAACCTCTTTCAT -3′

Hdm2-L54P-QC1: 5′-CTATGAAAGAGGTTCcTTTTTATCTTGGCCA-3′

Hdm2-L54P-QC2: 5′-TGGCCAAGATAAAAAgGAACCTCTTTCATAG-3′

Hdm2-Y48C-QC1:5′-CACAAAAAGACACTTgTACTATGAAAGAGGT-3′

Hdm2-Y48C-QC2: 5′-ACCTCTTTCATAGTAcAAGTGTCTTTTTGTG-3′

Hdm2-K36E-QC1: 5′-AAGCCATTGCTTTTGgAGTTATTAAAGTCTG-3′

Hdm2-K36E-QC2:5′-CAGACTTTAATAACTcCAAAAGCAATGGCTT-3′

### Vector and HDM2 library construction

2ConA NterHDM2 PET22b was created via inverse PCR with primers Hdm2-Nter-noHA-R and HA-F on 2ConA HDM2 PET22b. Primers HDMM62A-1 and HDMM62A-2 were used in QuikChange mutagenesis on 2ConA NterHDM2 PET22b to create 2ConA NterHdm2-M62A PET22b. All of the above mentioned constructs additionally encode a C-terminal HA tag.

Error-prone PCR was carried out on both 2ConA NterHDM2 PET22b and 2ConA NterHDM2-M62A PET22b using petF2 and petR and the mutant genes re-amplified with Hdm2-NdeI and HArevBamHI. The libraries were then ligated into 2ConA PET22b via Nde1/BamHI sites and re-amplified with petF2 and petR to produce library amplicons with T7 promoter and ribosome binding site required for *in vitro* transcription/translation (IVT), as well as the 2ConA RE site located before the T7 promoter site. p53-PET22b was also amplified with petF2 and petR for IVT of wild-type 53.

PM2 resistant parental clones obtained from the selection were amplified with petF2 and petR to create amplicons for secondary assays. M62AC8 parental clone was amplified with infus-Mdm2-F and Infus-M62AC8-Nter-R for cloning by infusion into 2ConA HDM2 PET22b that was amplified with M62AC8Hdm2-Nter-F and petATG-R to create full length 2ConA HDM2 M62AC8 PET22b. The mutation M62A was also removed from 2ConA HDM2 M62AC8 PET22b or 2ConA NterHDM2 M62AC8 PET22b via mutagenesis with M62AC8-revertQC1 and M62AC8-revertQC2. Single mutant HDM2 clones were also generated by Quikchange mutagenesis of 2ConA HDM2 PET22b or 2ConA NterHDM2 PETb using appropriate primer pairs. The same primers were used to introduce mutations into the parental pCMV-HDM2 mammalian expression construct.

### *In vitro* selection of HDM2 variants resistant to PM2

IVT reactions consisting of 0.5μM ZnCl_2_, 10μM PM2, p53 (10ng in preselection round, 10ng in round 1, 4ng in round 2, 2ng in rounds 3/4), library amplicons (5ng in preselection round, 5ng in round 1, 2ng in round 2, 1ng in round 3/4) in a total volume of 50μL PURExpress® *in vitro* protein synthesis solution (New England Biolabs) were assembled on ice and emulsified as previously described [50, 51]. After incubation at 37°C, the reactions were centrifuged at 8000rpm for 10mins to separate the aqueous and oil phase. The oil phase was removed and 50uL PBS was added to the pellet of aqueous phase compartments. The compartments were disrupted by four rounds of hexane extraction and the aqueous phase incubated with anti-HA antibody-coated protein G beads (Invitrogen) at 4°C with rotation. During round 4, PM2 (1μM) was added during this step to increase selection stringency. The beads were washed thrice with PBST-0.1%BSA, and thrice with PBS. The beads were resuspended in 20μl water and the protein-protein-DNA complexes eluted by incubation at 95°C for 5mins. The eluates were amplified with petF3 and HArevBamHI during a primary amplification and with Hdm2-NdeI and HArevBamHI during a secondary amplification. The products were cloned back into 2ConA-PET22b via Nde1/BamHI sites and re-amplified with petF2 and petR for the next round of selection.

### Secondary co-immunoprecipitation assay and western blot analysis

Protein G beads were incubated with anti-HA (1μg per 10μL beads) for 1 hour in PBST-3%BSA and subsequently washed thrice in PBST-0.1%BSA. IVT-expressed protein was incubated with the beads on a rotator for 30 mins. PM2 was added at 100μM and incubation carried out for 30 mins. IVT-containing secondary protein was added to the mixture and incubation allowed for 1 hour. Beads were finally washed thrice in PBST-0.1%BSA and thrice with PBS, and bound proteins eluted by resuspension in 20μL SDS-PAGE loading buffer and incubation at 95^°^C for 5 minutes. Where required, blank IVT extract (no template DNA added) was used as control. The eluates were subjected to electrophoresis, transferred to nitrocellulose membranes and probed for p53 with DO1-HRP or for HDM2 with anti-HA antibody followed by rabbit anti-mouse (Dakocytomation). Image acquisition was carried out using either film or digitally (Odyssey FC, Li-Cor). Un-cropped blot images are shown in Figure S5.

### Cell culture and reporter assay

Mouse embryonic fibroblast p53/Mdm2 double-knockout (DKO) cells (a kind gift from Guillermina Lozano) [58] were maintained in Dulbecco's modified Eagle's medium (DMEM) with 10% (v/v) foetal calf serum (FCS) and 1% (v/v) penicillin/streptomycin. The cells were seeded at 1.0 × 10^5^ cells/well in 6-well plates, 24 hours prior to transfection. Cells were co-transfected with parental or individual PM2-resistant HDM2 plasmid, p53-pcDNA plasmid, LacZ reporter plasmid and luciferase transfection efficiency plasmid using TurboFect transfection reagent (Thermo Scientific) according to the manufacturer's instructions. Nutlin and PM2 were added to selected wells at required concentrations 4.5 hours post-transfection. In all cases, the total amount of plasmid DNA transfected per well was equilibrated by addition of the parental vector pcDNA3.1a (+).

### β-Galactosidase assay and western blot analysis

DKO cells were harvested 24hours post transfection and β-galactosidase activities were assessed using the Dual-light System (Applied Biosystems) according to the manufacturer's protocol. β-galactosidase activity was normalized with luciferase activity for each sample. To check for expression levels of relevant proteins via western blot, 5 μg of the cell lysates were probed for p53 with horseradish peroxidase conjugated DO1 antibody, for HDM2 and actin with anti-HA antibody and AC15 antibody respectively followed by rabbit anti-mouse.

### Protein expression and purification

HDM2 (amino acids 1–125) was cloned as a GST-fusion protein using the pGEX-6P-1 GST expression vector (GE Healthcare). QuikChange site directed mutagenesis (Stratagene) was used to create the mutant HDM2-F55L (amino acids 1–125). The constructs were then transformed into *Escherichia coli* BL21(DE3) pLysS (Invitrogen) competent cells. Cells were grown in LB medium at 37°C and induced at OD_600 nm_ of 0.6 with 0.5 mM IPTG at 16°C. The cells were harvested by centrifugation after overnight induction, resuspended in binding buffer (50 mM Tris-HCl pH 8.0, 150 mM NaCl), and lysed by sonication. The lysate was centrifuged for 60 mins at 19,000xg at 4°C and applied to a 5 mL GSTrap FF column (GE Healthcare) pre-equilibrated in wash buffer (50 mM Tris-HCl pH 8.0, 150 mM NaCl, 1 mM DTT). On-column cleavage by PreScission protease (GE Healthcare) was carried out overnight at 4°C and the cleaved protein eluted off the column with wash buffer. Dialysis into buffer A solution (20 mM Bis-Tris, pH 6.5, 1 mM DTT) using HiPrep 26/10 Desalting column was performed and the protein sample was subsequently loaded onto a cation-exchange Resource S 1 mL column (GE Healthcare) pre-equilibrated in buffer A. Six column volumes of buffer A was used to wash the column and the bound protein was eluted with a linear gradient in buffer comprising 1 M NaCl, 20 mM Bis-Tris pH 6.5, and 1 mM DTT over thirty column volumes. Protein purity as assessed by SDS-PAGE was ~95%, and the proteins were concentrated using Amicon-Ultra (3 kDa MWCO) concentrator (Millipore).

### Fluorescence polarization (FP) assay

Fluorescence anisotropy assays were performed as previously described [3]. Titrations of purified wild-type and mutant HDM2 (1–125) were incubated with 50 nM of carboxyfluorescein (FAM) labelled 12-1 peptide (FAM-RFMDYWEGL-NH2) to determine the dissociation constants for the peptide-protein interaction. Apparent K_d_s of PM2 and human p53 peptide (Ac-QETFS DLWKLLPEN–NH2) were then determined by competitive fluorescence anisotropy. Titrations of PM2 and human p53 peptide were carried out with a constant concentration of wild-type HDM2 at 150 nM, mutant HDM2-F55L at 200 nM and the labelled peptide at 50 nM. Anisotropy measurements were carried out using the Envision Multilabel Reader (PerkinElmer). All experiments were carried out in PBS (2.7 mM KCl, 137 mM NaCl, 10 mM Na_2_HPO_4_ and 2 mM KH_2_PO_4_, pH 7.4), 3% DMSO and 0.1% Tween-20 buffer. All titrations were carried out in duplicate (n = 3 to 9 independent titrations). Curve fitting was carried out using Prism 4.0 (GraphPad).

### F2H co-localization assay

Plasmids encoding the GFP-tagged bait p53 (amino acids 1-81) fusion protein and different RFP-tagged prey HDM2 (amino acids 7-134) fusion proteins were co-transfected into transgenic F2H-BHK cells (F2H-Kit Basic, ChromoTek GmbH) [59] in 96 multiwell plates (μClear Greiner Bio-One, Germany) using the Lipofectamine 2000 (Life Technologies) reverse transfection protocol according to manufacturer's instructions with 0.2 μg DNA and 0.4 μl Lipofectamine 2000 per well. 16 hours after transfection, cells were treated with Nutlin (0-10 μM) or PM2 (0-50 μM) for 6-8 hours in serum-free DMEM at 37°C, 5% CO_2_. Interaction (%) was determined as the ratio of cells showing co-localization of fluorescent signals at the nuclear spot to the total number of evaluated cells. The INCell Analyzer 1000 with a 20X objective (GE Healthcare) was used for automated image acquisition. Automated image segmentation and analysis was performed with the corresponding INCell Workstation 3.6 software. At least 100 co-transfected cells were analyzed per well. Titrations were carried out independently (n=2).

### Molecular modelling

To model the interactions of the N terminal domain of human HDM2 with the peptides (p53/stapled PM2) and Nutlin, the crystal structures of the HDM2-p53 complex [60] (PDB code 1YCR, resolved at 2.6Å), the crystal structure of the stapled peptide M06 bound to HDM2-M62A (PDB code 4UMN, resolved at 1.99 Å) [47] and the HDM2-Nutlin complex [61] (PDB code 1RV1, resolved at 2.3Å) were used.

## SUPPLEMENTARY FIGURES




