# Supplementary material for: Avoiding drug resistance through extended drug target interfaces: a case for stapled peptides
Source: Oncotarget. 2016 Apr 4;7(22):32232–46. doi: 10.18632/oncotarget.8572 (PMC5078010; doi:10.18632/oncotarget.8572)
Supplement: Supplementary file 1 [file oncotarget-07-32232-s001.pdf]

Avoiding drug resistance through extended drug target interfaces: a case for stapled peptides

SUPPLEMENTARY FIGURES

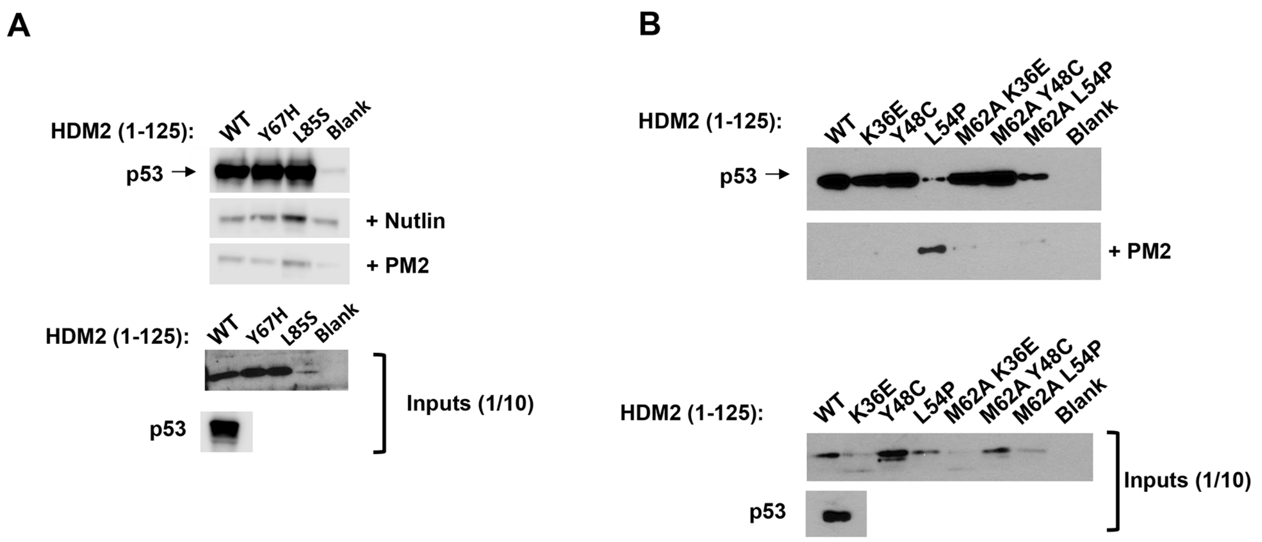

Supplementary Figure S1: Selected HDM2 mutants do not display significant *in vitro* PM2-resistance phenotype. A. *In vitro* pull-down assay showing no significant reduction in PM2 inhibition for the WT and indicated C8-derived HDM2 (1-125) point mutants. Blank indicates background p53 binding in absence of HDM2. Note: exposure time for HDM2 inputs is 8 hours (developed using film) and 30 seconds for all other panels (digitally acquired). **B.** *In vitro* pull-down assay showing no significant reduction in PM2 inhibition for the WT and indicated C11/C12-derived HDM2 (1-125) mutants. Note: exposure time for HDM2 inputs is 8 hours, 10 seconds for all other panels except for +PM2 (10 minutes). All blots developed using film.

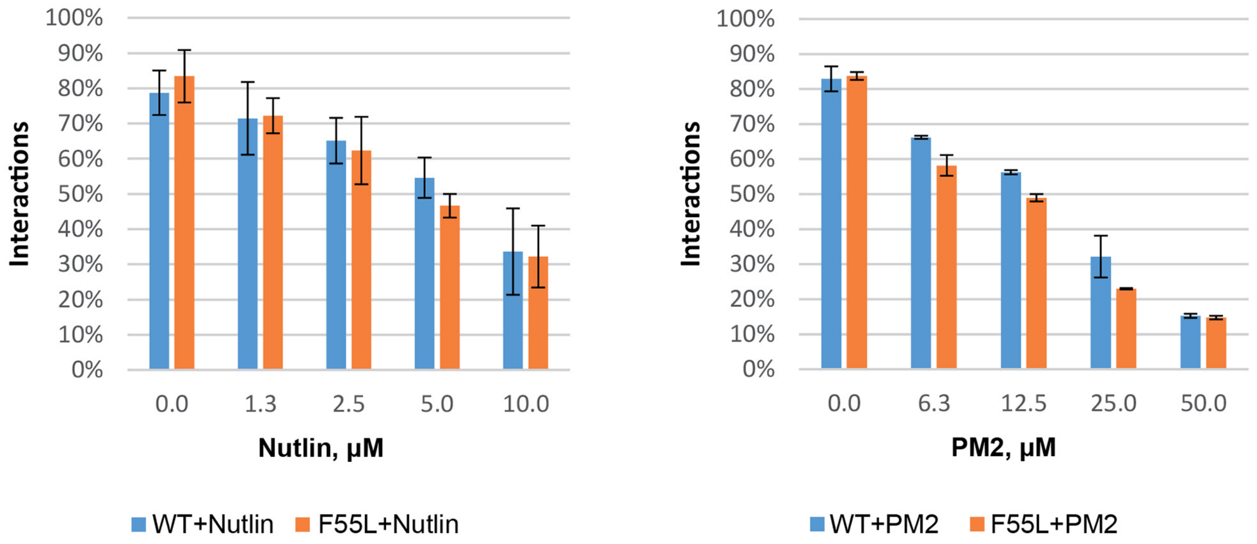

Supplementary Figure S2: Fluorescent 2-hybrid assay does not show any significant difference in PM2 ability to disrupt p53 interaction with WT HDM2 versus F55L HDM2 mutant. Titration of Nutlin (left) or PM2 (right) does not result in increased dissociation of F55L-p53 complex (orange bars) compared to WT-p53 complexes (blue bars). Bar chart shows mean interaction values and range from two independent experiments (at least 100 cells evaluated per data point).

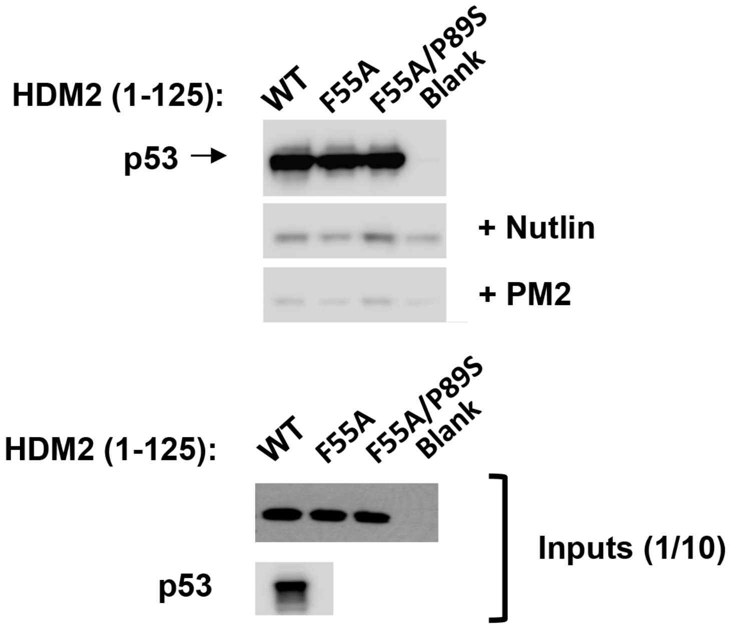

Supplementary Figure S3: Selected HDM2 single and double point mutants do not display in vitro PM2-resistance phenotype. *In vitro* pull-down assay showing no significant reduction in PM2 inhibition for the WT and indicated HDM2 (1-125) single and double point mutants. Blank indicates background p53 binding in absence of HDM2. Note: exposure time for HDM2 inputs is 8 hours (developed using film) and 1 minute for all other panels (digitally acquired).

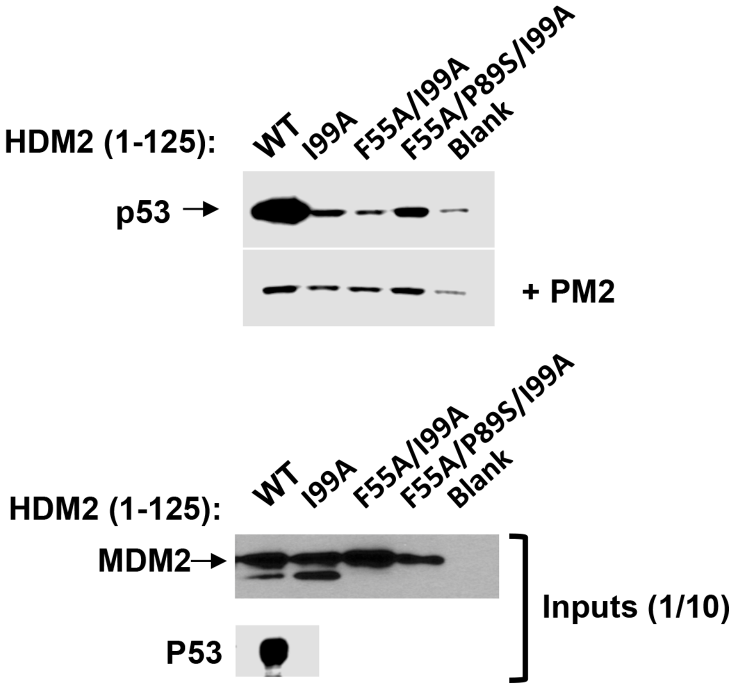

Supplementary Figure S4 : The I99A mutation in HDM2 (1-125) significantly ablates p53 binding. *In vitro* pull-down assay showing significant reduction in p53 binding to HDM2-I99A and indicated mutants. Blank indicates background p53 binding in absence of HDM2. Note: exposure time for HDM2 inputs is 8 hours (developed using film) and 5 minutes for all other panels (digitally acquired).

Supplementary Figure S5: Un-cropped blot images.

See Supplementary File 1
