# Supplementary material for: Avoiding drug resistance through extended drug target interfaces: a case for stapled peptides
Source: Oncotarget. 2016 Apr 4;7(22):32232–46. doi: 10.18632/oncotarget.8572 (PMC5078010; doi:10.18632/oncotarget.8572)

Figure S5 : Uncropped blot images

Figure 2A:

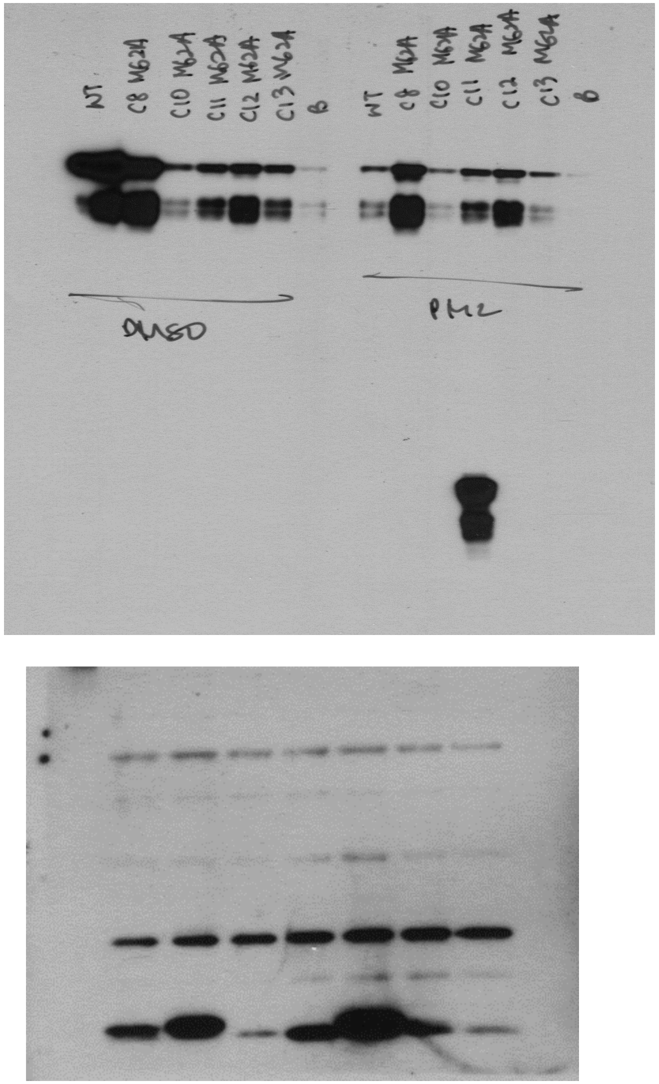

Figure 2B:

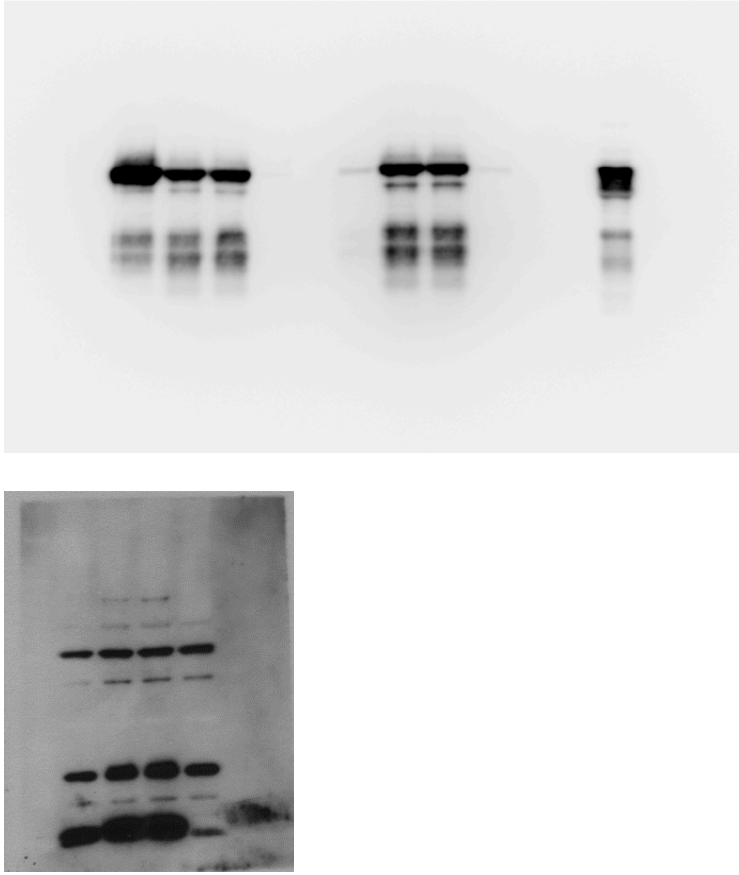

Figure S5 : Uncropped blot images

Figure 4A:

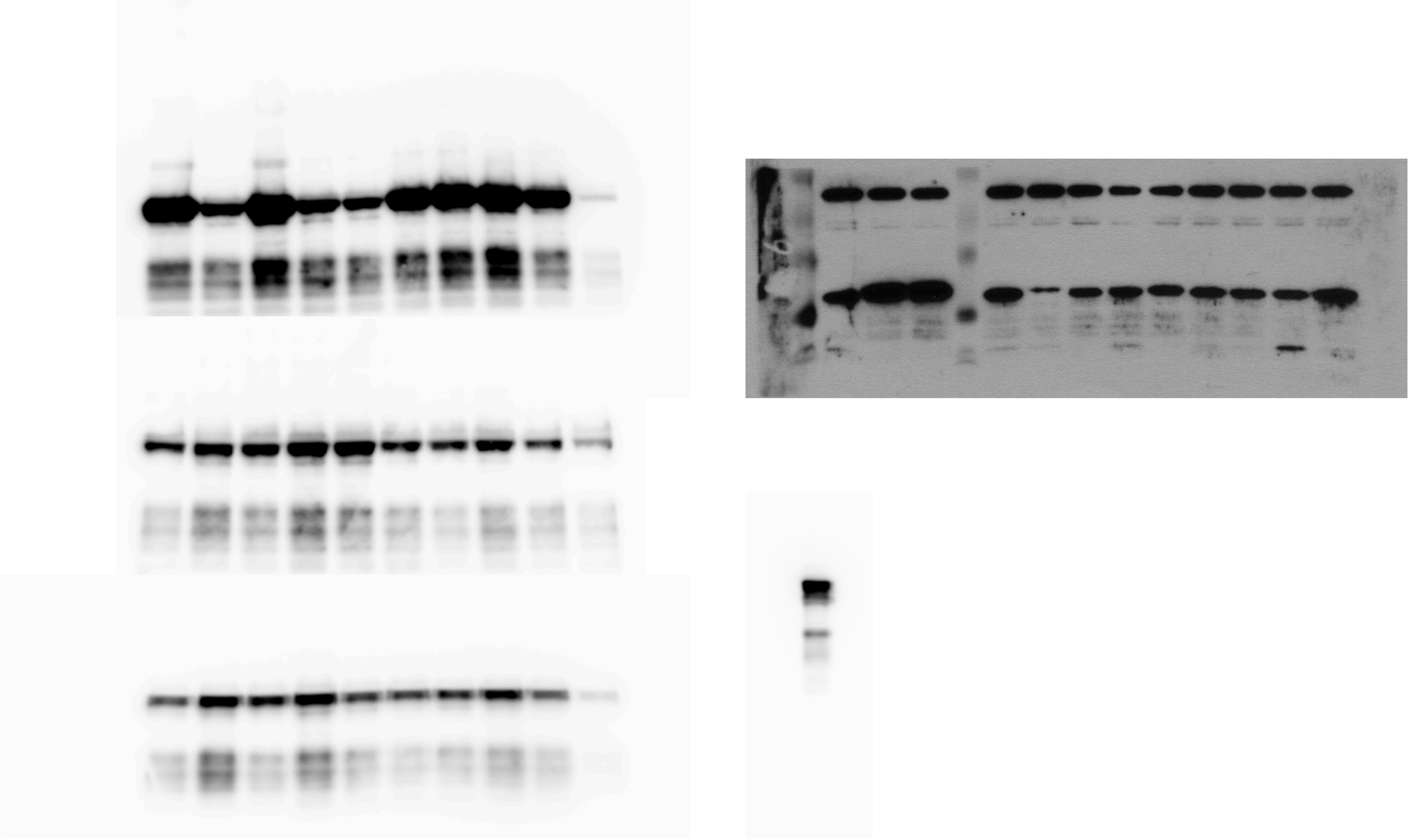

Figure S5 : Uncropped blot images

Figure 4B:

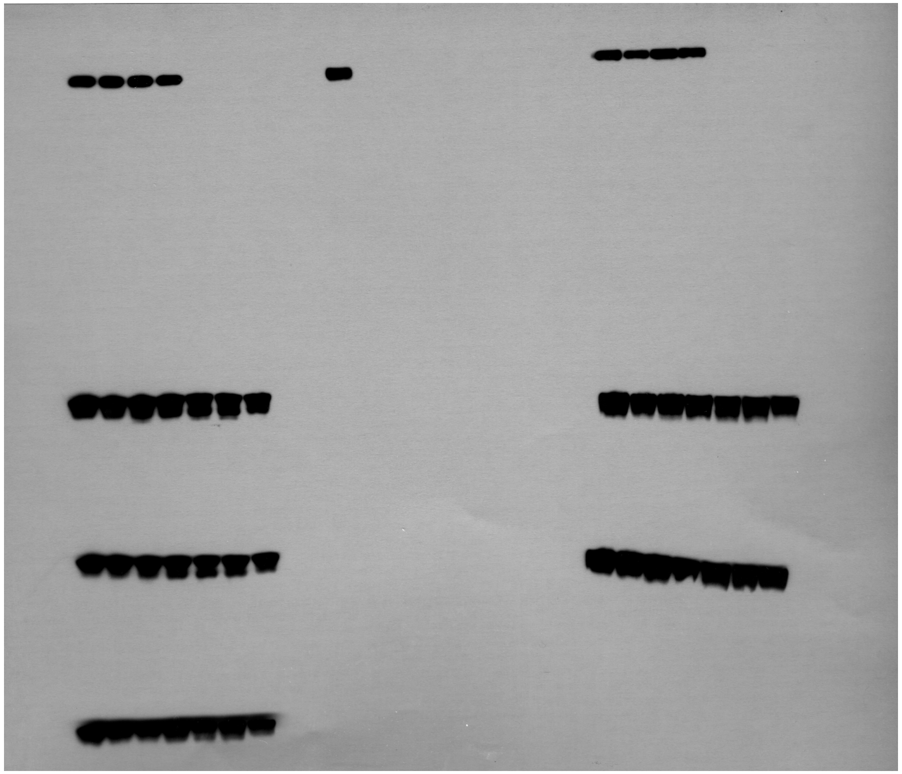

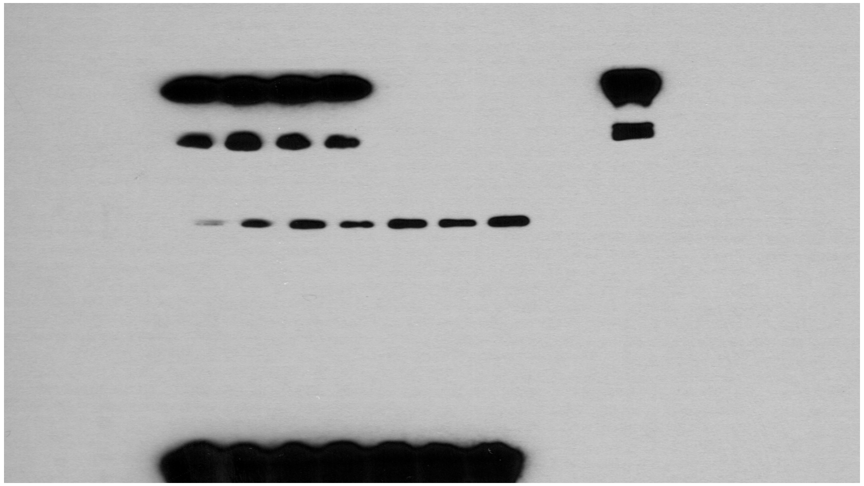

Figure S5 : Un-cropped blot images

Figure 5A

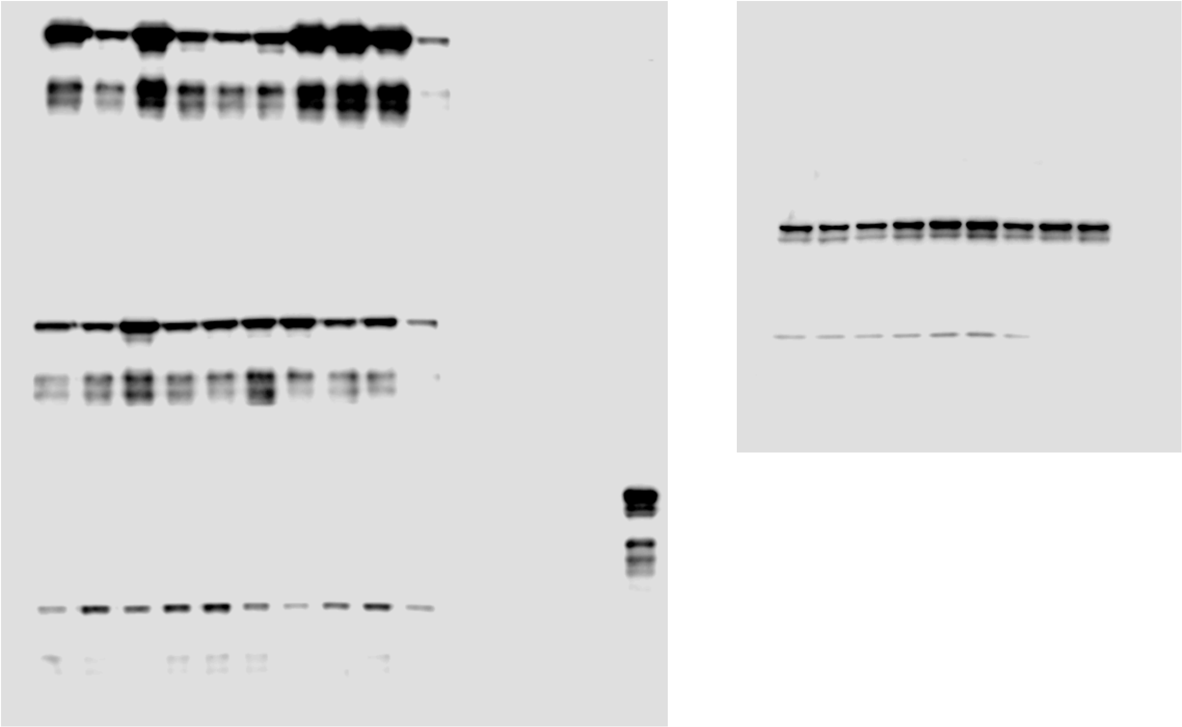

Figure S5 : Uncropped blot images

Figure 5B:

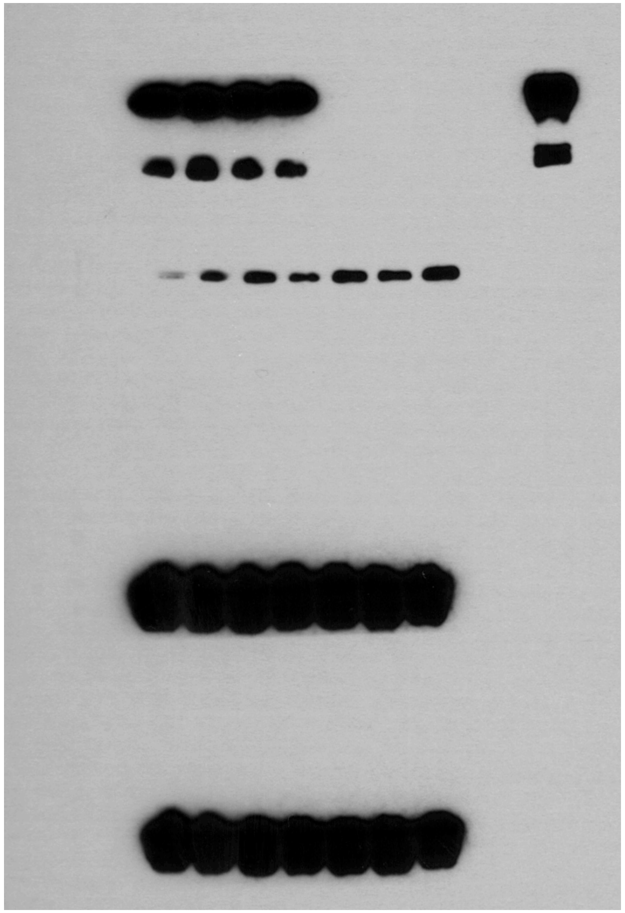

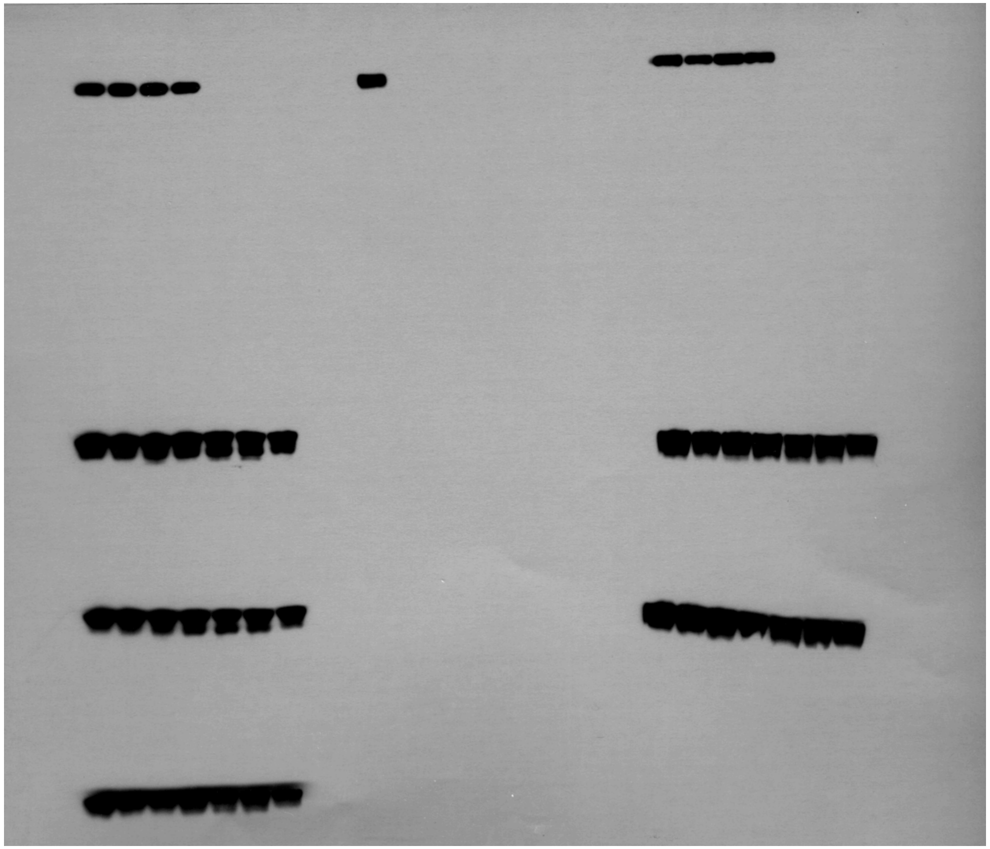

Figure S5 : Uncropped blot images

Figure 6A:

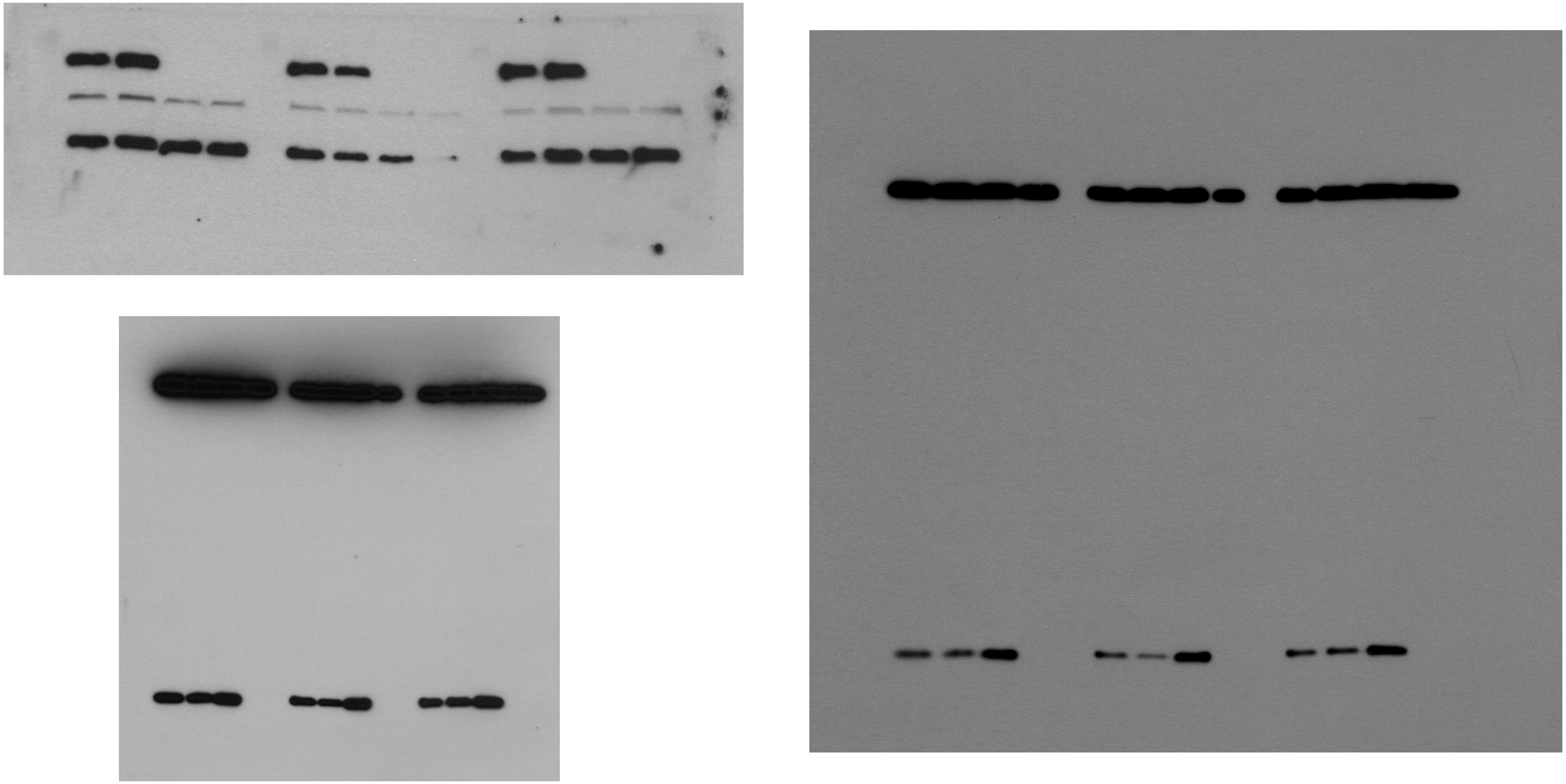

Figure 6B:

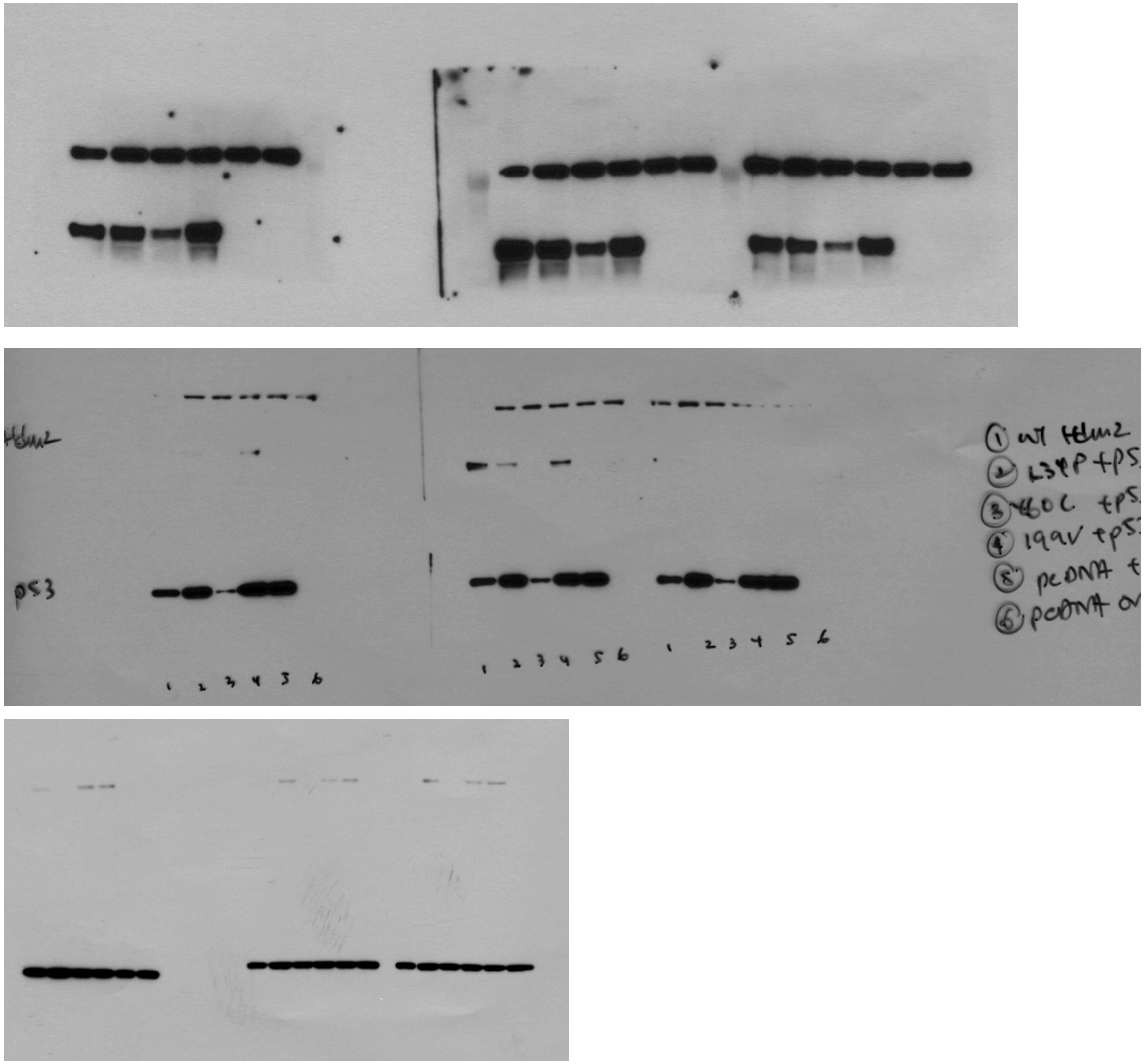
Hdm2
p53
1 2 3 4 5 6
1 2 3 4 5 6 1 2 3 4 5 6
1 wt Hdm2
2 L34P +p5
4 I99V +p5
pcDNA +
6 pcDNA

Figure S5 : Uncropped blot images

Figure 6C:

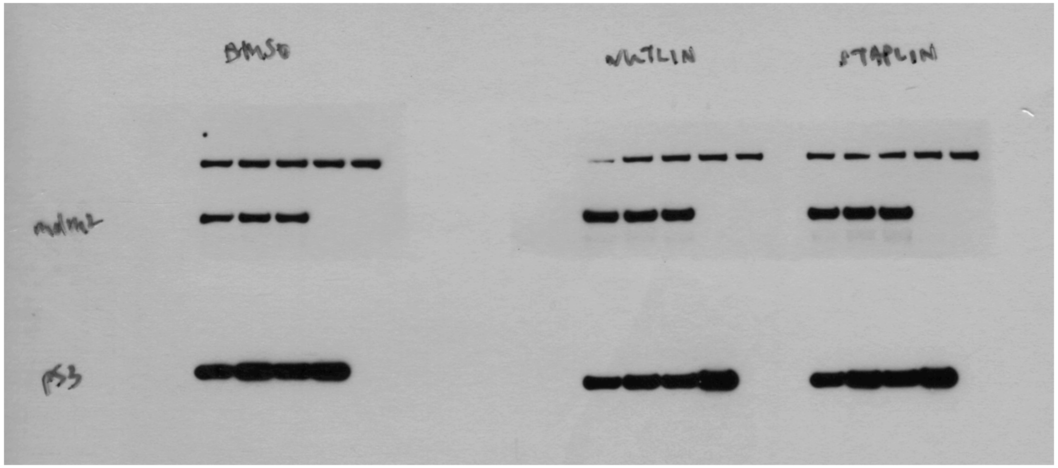

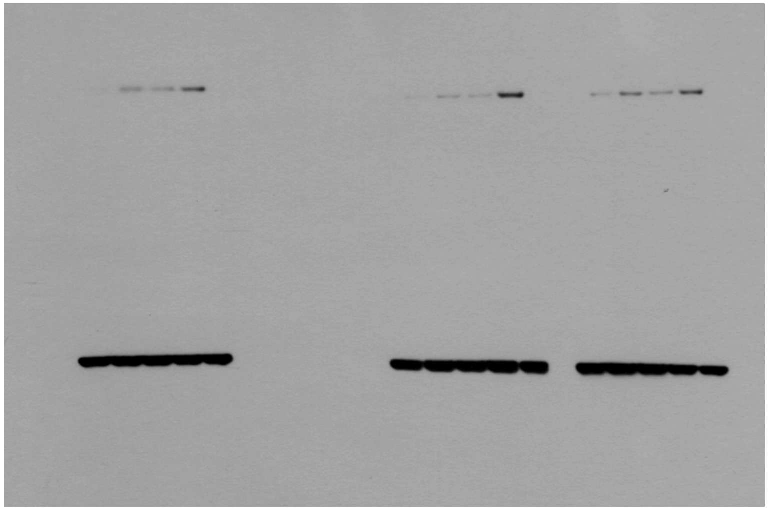

Figure S5 : Uncropped blot images

Figure S1-A:

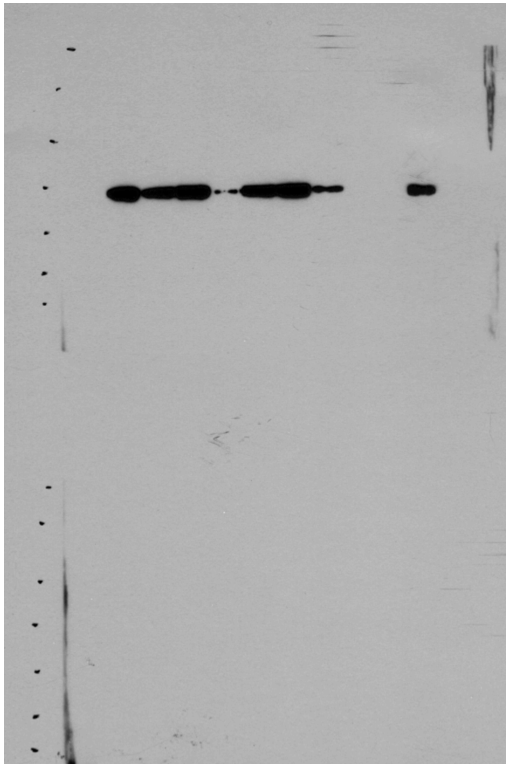

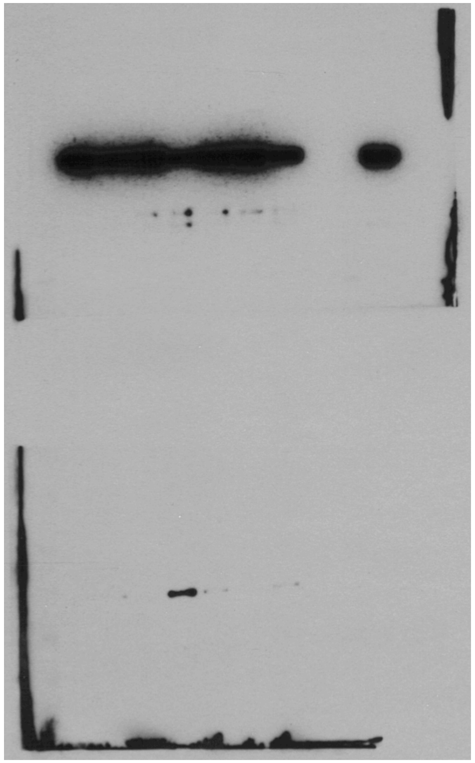

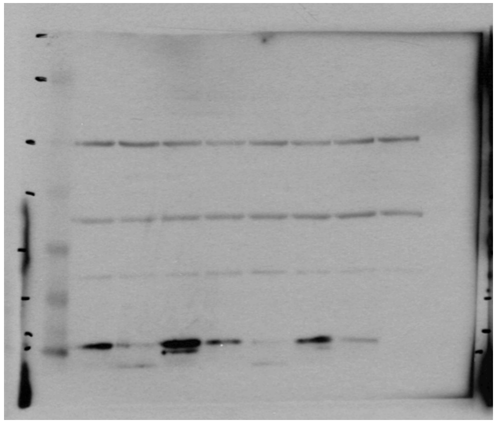

Figure S5 : Uncropped blot images

Figure S1-B:

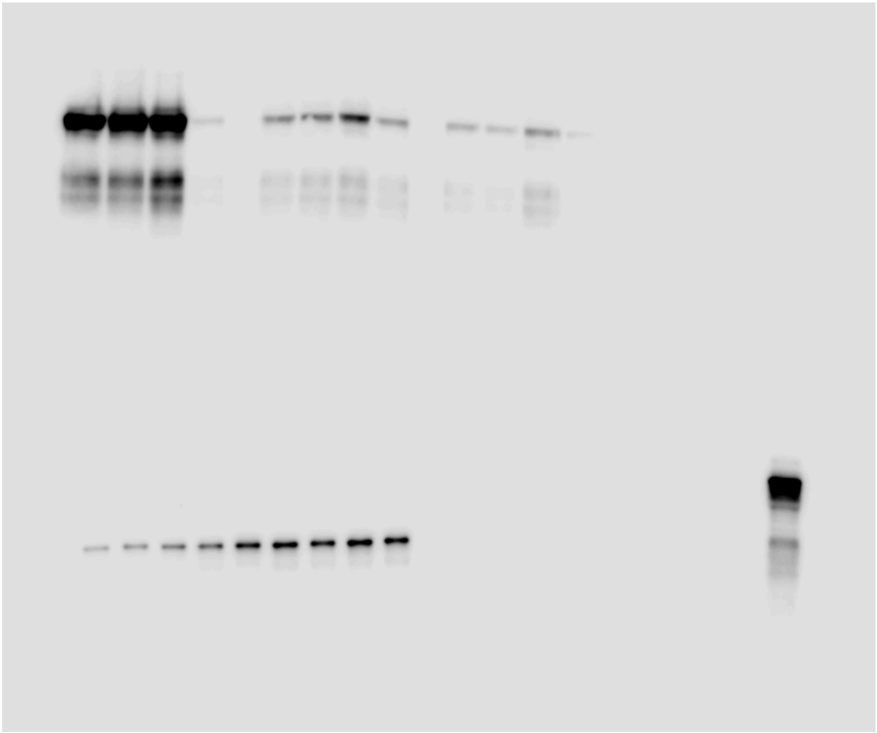

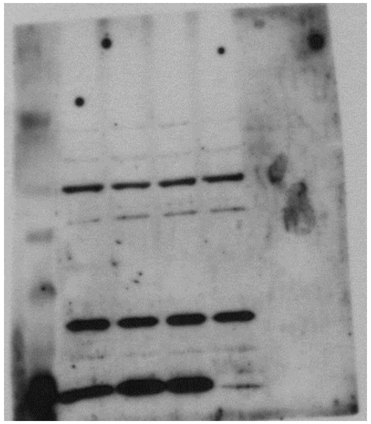

Figure S5 : Uncropped blot images

Figure S3:

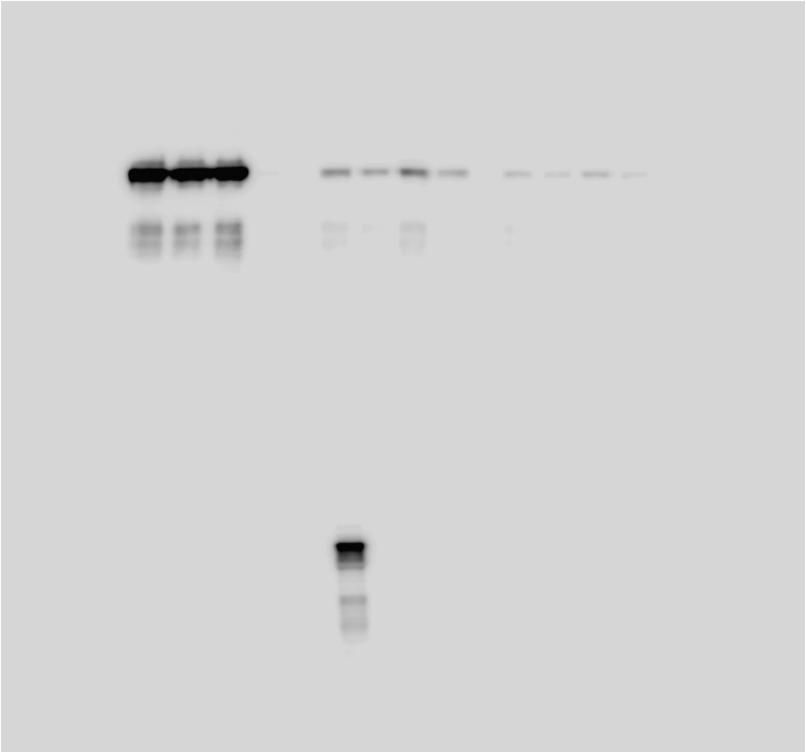

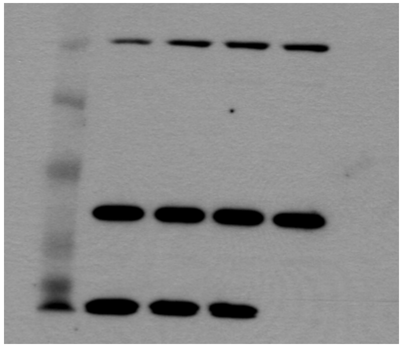

Figure S5 : Uncropped blot images

Figure S4:

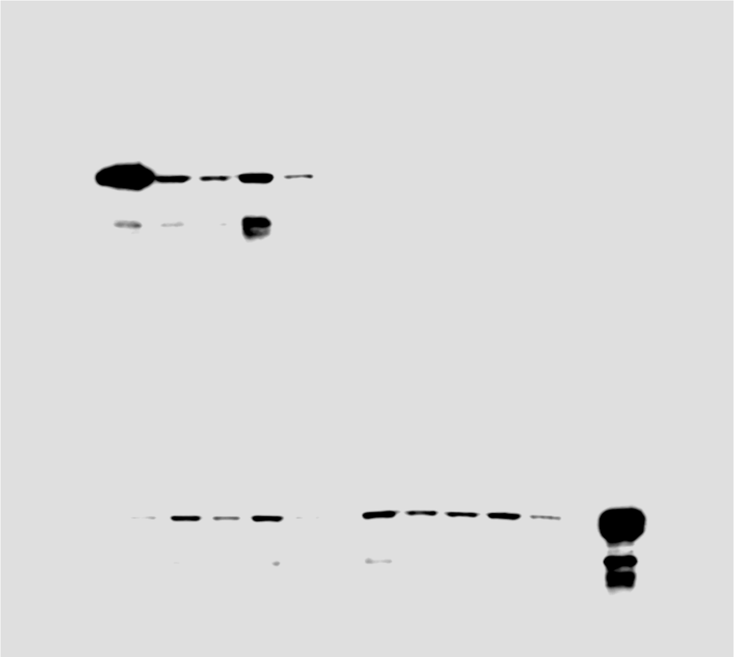

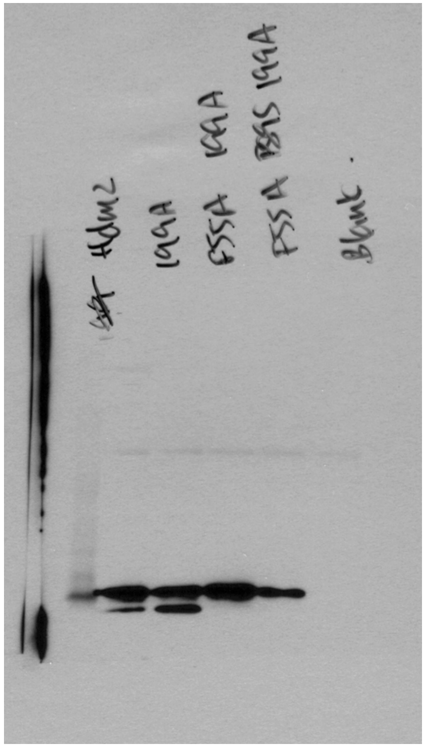

Supplement: Supplementary file 2 [file oncotarget-07-32232-s002.pdf]
